# Developing a Cognitive Behavioural Therapy for Insomnia Intervention for Adolescents With Co‐Morbid Mental Health Using an Iterative Expert Consultation Process

**DOI:** 10.1111/jsr.70174

**Published:** 2025-08-20

**Authors:** Stephanie McCrory, Megan Crawford, Kenneth MacMahon, Carey Ross, Dipalika Mohanty, Dinaish Mistry, Anastasia Thalia Fulton Chadwick, Leanne Fleming

**Affiliations:** ^1^ Strathclyde Sleep Centre University of Strathclyde Glasgow UK; ^2^ NHS Psychological Services Ayrshire & Arran UK

**Keywords:** adolescents, CBTi, insomnia, mental health

## Abstract

Insomnia is prevalent in adolescents with co‐morbid mental health problems but is often overlooked due to limited access to training for practitioners in the assessment and treatment of insomnia. Whilst Cognitive Behavioural Therapy is the recommended treatment for insomnia in adults (CBTi), there are no standard treatment guidelines for adolescents and limited research with adolescents with co‐morbid mental health problems. Therefore, our aim was to develop a CBTi intervention for adolescents with co‐morbid mental health problems. This study utilised an iterative expert consultation approach to develop a CBTi intervention and define the appropriate target population, components and delivery. Eighteen experts were identified from literature searches and professional networks and invited to participate. Three iterative rounds of questionnaires were conducted and included both open‐ended and closed‐ended questions. In total, seven experts participated (R1 = 7, R2 = 5 and R3 = 1). In R1, four main themes emerged: (1) CBTi is appropriate for early–mid adolescents with anxiety/depression, (2) the proposed content and format were appropriate but required adaptation, (3) the proposed method of delivery was appropriate (i.e., in‐person, by trained practitioner) and (4) parent/caregiver involvement is necessary. In R2, the intervention protocol was reviewed and finalised. In R3, the intervention materials were reviewed. The newly developed intervention comprises 4 weekly sessions, intervention materials and a training package for non‐sleep experts. To our knowledge, this is the first study to utilise an iterative expert consultation process to develop an insomnia intervention for adolescents with co‐morbid mental health.

## 1 Introduction

Research reports that up to 30% of adolescents meet criteria for insomnia disorder (ID) (de Zambotti et al. 2018; Falch‐Madsen et al. 2020; Fernandez‐Mendoza et al. 2021; Hysing et al. 2013). Insomnia is defined as dissatisfaction with sleep quality and/or quantity, despite adequate sleep opportunity. This includes persistent difficulty with sleep initiation, maintenance, or early‐morning awakening that results in distress or impairment in daytime functioning (APA 2013). Insomnia in adolescents is a significant public health concern, particularly as insufficient sleep is associated with poorer academic performance, impaired cognitive functioning, increased risk‐taking and mental and physical health problems (de Zambotti et al. 2018; Uccella et al. 2023; Zhang et al. 2022).

The evolution of chronic insomnia is outlined by the 3P model (Spielman et al. 1987). This model describes the development and maintenance of insomnia via interactions between predisposing, precipitating and perpetuating factors. Adolescents are particularly vulnerable to insomnia due to changes in their circadian and homeostatic bioregulatory processes that result in altered sleep timing and duration (Carskadon 2011; Falch‐Madsen et al. 2021; Kahn 2023). When these changes are accompanied by precipitating factors, such as stressful life events, acute sleep disturbance can arise (Ellis et al. 2021). Adolescents may then engage in behaviours to counteract this acute sleep disturbance, such as extending time in bed on weekends and increasing technology use before bed. However, these maladaptive compensatory behaviours are known to perpetuate the problem and lead to chronic insomnia.

Rates of insomnia are higher in adolescents with comorbid mental health problems. Research demonstrates that up to 92% of adolescents with depression, and 88% with anxiety, report significant sleep disturbance (Brown et al. 2018; Reynolds et al. 2020; Short et al. 2020). Furthermore, up to 48% of adolescents with depression, and 32% with anxiety, meet criteria for insomnia using self‐report measures (Hysing et al. 2022; van Dyk et al. 2019). Some studies indicate a bidirectional relationship between sleep and mental health, and others indicate that insomnia has a causal role in the onset and development of mental health problems, including depression and anxiety (Hertenstein et al. 2019; Lovato and Gradisar 2014; Narmandakh et al. 2020; Scott et al. 2021). A recent meta‐analysis found that adolescents and young adults who experienced sleep problems had a 2‐fold risk of developing major mental health disorders, such as depression, bipolar or schizophrenia, before the age of 30 (Scott et al. 2021). Insomnia requires independent evaluation and treatment; otherwise, it can persist beyond remission of mental health problems and predicts relapse (Morin and Jarrin 2022; Palagini et al. 2022). Despite this, insomnia is rarely assessed or treated in adolescents.

Cognitive Behavioural Therapy for insomnia (CBTi) is a multi‐component intervention and the recommended first‐line treatment for insomnia in adults (Edinger et al. 2021; Xu et al. 2021). CBTi typically comprises sleep restriction therapy (SRT), stimulus control (SCT), cognitive techniques, relaxation and sleep hygiene (SH) education. Several systematic reviews and meta‐analyses have demonstrated that CBTi is effective for insomnia in adults, with medium to large effect sizes (Edinger et al. 2021; Xu et al. 2021). Research has also highlighted additional therapeutic benefits of CBTi, including reduced symptoms of depression, anxiety, post‐traumatic stress disorder and improved quality of life (Blake et al. 2017; Hertenstein et al. 2022). Trials of CBTi in adolescents are more limited, but demonstrate significant improvements in SOL, duration and quality, as well as mental health outcomes (Blake et al. 2017; de Zambotti et al. 2018; Dewald‐Kaufmann et al. 2019).

Despite these promising outcomes, access to treatment remains limited. This is because there is a shortage of trained practitioners and limited access to CBTi training (Baglioni et al. 2020). In 2015, there were less than 800 qualified behavioural sleep medicine providers worldwide, and most were based in the United States (Thomas et al. 2016). Since then, there has been an effort to streamline CBTi education; however, access to training courses remains scarce and costly (Jernelöv and Blom 2023). Furthermore, there is an absence of standard treatment guidelines informing the implementation of CBTi protocols, specifically, the necessary adaptations for adolescents (Koffel et al. 2018). Eight studies have explored the implementation of CBTi within adolescent mental health services, where insomnia may be especially prevalent (Åslund et al. 2020; Bradley et al. 2018; Cliffe et al. 2020; Mathews et al. 2023; Orchard et al. 2020; Rollinson et al. 2021, 2024; Zetterqvist et al. 2021). However, in most of these studies (except Rollinson et al. 2021) the intervention was delivered by trained practitioners with expertise and experience in delivering CBTi. This limits the accessibility, scalability and implementation of CBTi in practice. Some protocols reported adaptations including additional psychoeducation on developmental sleep changes (Bradley et al. 2018; Rollinson et al. 2021, 2024; Zetterqvist et al. 2021), limiting electronic/social media use (Bradley et al. 2018; Zetterqvist et al. 2021) and milder SRT that focus on sleep scheduling and wake‐up times only (Bradley et al. 2018; Rollinson et al. 2021, 2024). However, in most of these studies (except Bradley et al. 2018; Rollinson et al. 2021, 2024), there was limited stakeholder engagement to develop the protocol and inform adaptations for this population.

Two recent reviews examining sleep and behavioural treatments for insomnia (Meltzer et al. 2021; Reynolds et al. 2023) highlighted key areas that require further investigation in the field of adolescent sleep. These include research with special populations (including those with comorbid mental health problems), treatment components, modes of delivery to increase access to treatment and family involvement. To address these gaps, our aim was to consult with appropriate experts to develop a CBTi intervention protocol for adolescents with insomnia and comorbid mental health problems within mental health services. Our specific objectives were to:

1. To define the target population.
2. To explore what CBTi components should be included.
3. To investigate the appropriate mode of delivery and family involvement.

## 2 Materials and Methods

### 2.1 Design

This study utilised an iterative expert‐consultation approach to develop a CBTi intervention protocol for adolescents with insomnia and comorbid mental health problems. The research team comprised two Behavioural Sleep Medicine experts (L.F. and M.C.), a PhD student (S.M.), four Masters students (C.R., D.M., D.M. and A.C.) and a Head of Service from a local Child and Adolescent Mental Health Service (CAMHS). Development of the consultation protocol included identifying potential expert participants, reviewing and approving questionnaires for data collection, conducting data analyses, finalising the intervention protocol and materials.

### 2.2 Participants

This study utilised a purposeful sampling method to recruit an international panel of academics and practitioners with expertise in adolescent behavioural sleep medicine or adolescent mental health. All participants were educated to post‐graduate level (either doctorate or PhD) and were identified from research outputs or clinical practice as having relevant expertise and experience. Taking into consideration the aims of the research and the timeframe, we aimed to recruit up to 12 participants, with an anticipated dropout of 20% between each round (Chalmers and Armour 2019). In total, 18 experts were contacted and invited to participate.

### 2.3 Procedure and Materials

Ethical approval for this study was obtained from the University of Strathclyde, Department of Psychology Ethics Committee. Data collection took place between August 2022 and June 2023. All potential participants were contacted via email invitation (all contact details were publicly available) that contained a description of the study aims, relevant background information and a link to an online consent form and questionnaire. Potential participants were sent two reminder emails if they had not responded within 2 weeks. If no response was received following these reminder emails, their contact details were removed from the mailing list. Only participants who completed the first‐round questionnaire were contacted to participate in subsequent rounds.

A predetermined consensus threshold of 80% was selected as appropriate, mirroring Delphi guidelines (King et al. 2021). In cases where no consensus or conflicting opinions were observed, the research team discussed to determine the appropriate outcome. To maintain anonymity and reduce the risk of bias, an independent researcher was responsible for retrieving and anonymising participant responses. Responses were included if participants completed ≥ 20% of the questionnaire.

### 2.4 Questionnaires

**FIGURE 1:**
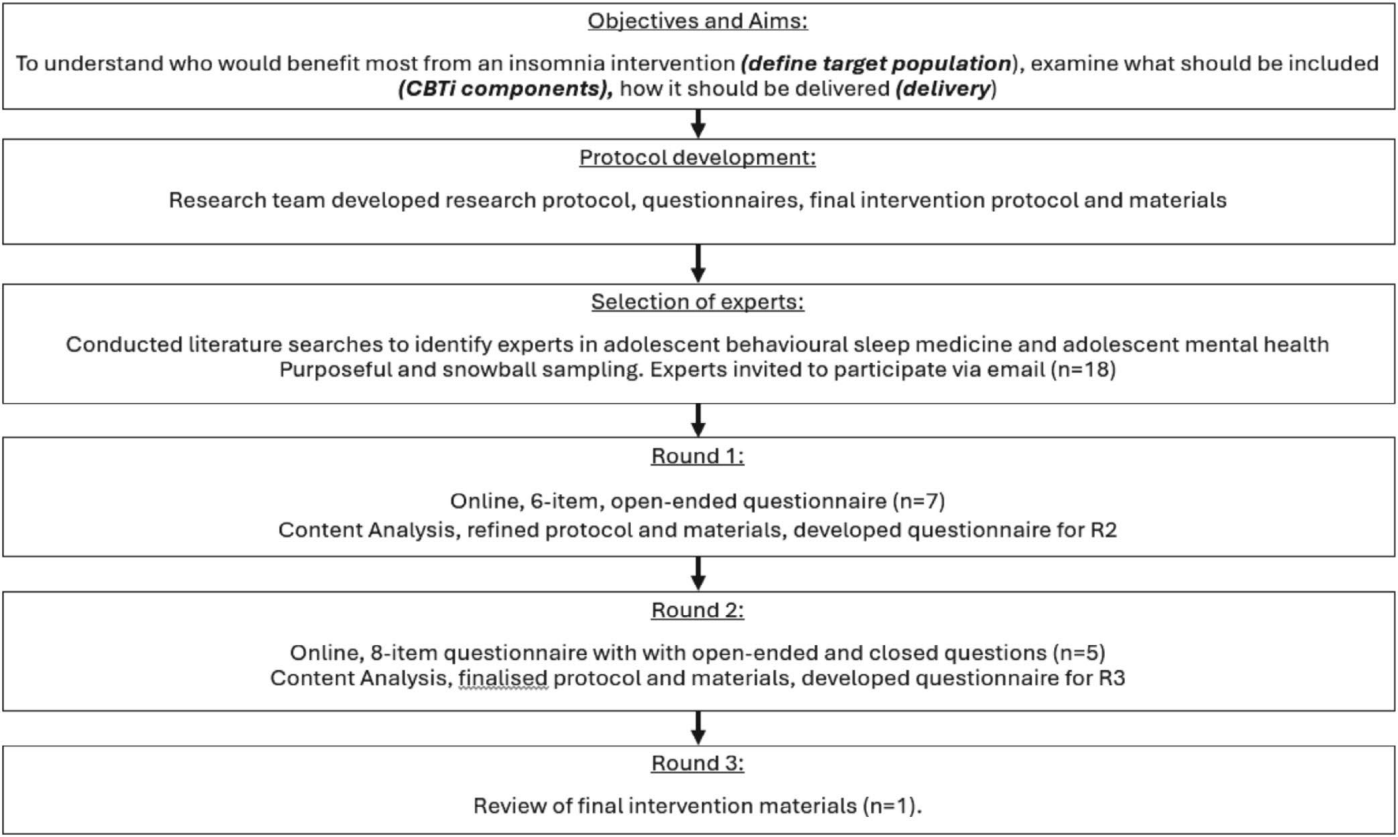
Study overview.

The data was collected via three iterative rounds of questionnaires. The aim of the questionnaires was to define the target population and assess the appropriateness of the content and method of delivery to ensure it was suitable for adolescents with insomnia with comorbid mental health problems (see Supporting Information). Only the first questionnaire (round 1, R1) collected demographic information including age, gender, occupation, level of education, area of expertise and geographical location. Questionnaires were distributed online via Qualtrics, and participants could skip the content‐related question if they felt that they lacked the necessary knowledge to provide feedback. See Figure 1 for an overview of the study.

### 2.5 Round 1 (R1)

**FIGURE 2:**
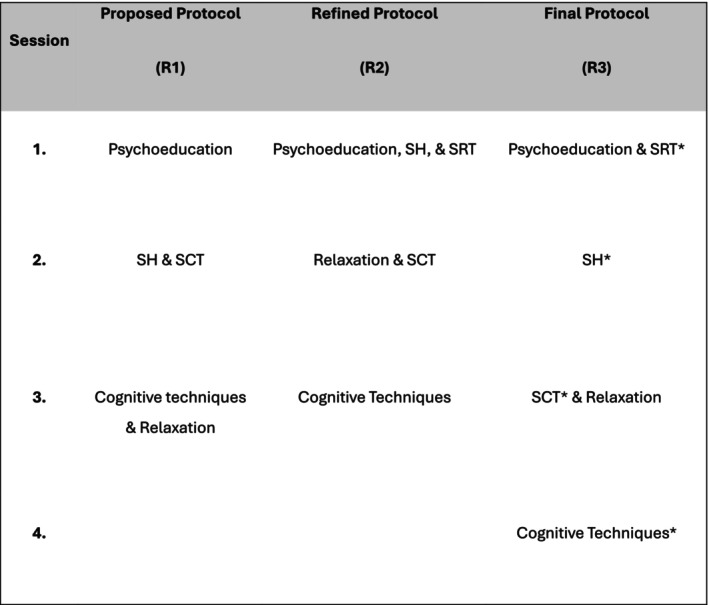
Overview of intervention protocols. SCT, stimulus control therapy; SH, sleep hygiene; SRT, sleep restriction therapy. *SRT instructions reviewed in each session.

The purpose of R1 was to use an exploratory approach to obtain detailed qualitative feedback on who the intervention would be appropriate for, what should be included in the intervention, and how the intervention should be delivered. Participants were first asked to provide feedback on the target population, specifically, ‘Who would benefit most from a targeted intervention to improve adolescent sleep and comorbid mental health? (please consider age, mental and physical health, additional needs and any other relevant characteristics)’. Then, a summary of a proposed CBTi protocol was presented, based on American Academy of Sleep Medicine guidelines (Edinger et al. 2021) (see Figure 2), and participants were asked to ‘comment on the structure and content of the sleep intervention and whether you feel it is appropriate for adolescents with comorbid sleep and mental health problems?’. They were then asked to identify if they felt any content was missing. The participants were asked to respond to ‘what is the most appropriate method of delivery of a sleep intervention for adolescents with comorbid mental health problems’. Participants were encouraged to consider the number and length of sessions, time in between sessions, inclusion of a booster session, session facilitator and any other aspects of delivery. Finally, the participants were asked to respond ‘yes’, or ‘no’ to indicate if they felt parent/carer involvement was necessary, and to provide feedback on the nature of involvement. Following completion of R1, the data was downloaded and anonymised by the independent researcher. The findings were analysed by five members of the research team (S.M., D.M., D.M., C.R. and A.C.) using content analysis (Hsieh and Shannon 2005; Krippendorff 2018). A preliminary codebook was created; all five reviewers then coded the data independently and met to discuss and resolve any conflicts. Following initial coding, the research team met to identify, refine and define themes.

### 2.6 Round 2 (R2)

The questionnaire for R2 was developed based on the feedback from R1 and was designed to follow‐up on key uncertainties that emerged. R2 also included an updated intervention protocol (see Figure 2) based on the recommendations from R1. The questions included in R2 were more specific and focused on the comorbidities that may not be suitable for CBTi interventions, the specific components of CBTi to be included, and additional questions about the mode of delivery. Following completion of R2, the data was downloaded and analysed using frequency statistics for closed‐ended questions and content analysis (as described above) for open‐ended questions. The findings from R2 were used to finalise the protocol and develop the intervention materials for R3.

### 2.7 Round 3 (R3)

In R3, participants were asked to review the final intervention materials and to indicate whether the materials were (a) appropriate, (b) required additional amendments or (c) not appropriate, and encouraged to provide additional feedback or comments. See Figure 2 for an overview of the amended intervention protocol.

## 3 Results

### 3.1 Demographics

In total, there were 18 responses in R1. However, 11 of those responses were incomplete (≤ 20% of the questionnaire had been completed); therefore, seven responses were included in R1. The same participants who completed R1 were invited to participate in R2. In R2, five participants responded and were contacted to participate in R3. One participant reviewed the final intervention materials in R3. Participant demographics for each round are presented in Table 1. Participants were mostly female, based in Europe and there had expertise in both adolescent sleep medicine and mental health. All participants had obtained their PhD for a minimum of 2 years (*m* = 9.71 years, SD = 6.74) and had contributed as authors on publications ranging from 9 to > 100 articles.

**TABLE 1:** Participant demographic data.

		R1	R2	R3
		*N* = 7	*N* = 5	*N* = 1
Age	25–34 years	2	2	1
	35–44 years	2	1	
	45–54 years	3	2	
Gender	Female	5	4	1
Location	Europe	6	4	1
	North/Central America	1	1	
Expertise	Adolescent Behavioural Sleep Medicine	3	1	
	Adolescent Mental Health	2	3	1
	Both	2	1	
Occupation	Academic	6	4	1
	Practitioner			
	Both	1	1	

### 3.2 Round 1 (R1)

From R1, four themes were generated from the data. See Table 2 for a summary of the main themes and supporting quotes.

**TABLE 2:** Themes and quotations.

Theme	Quotations
Sleep intervention is appropriate for early‐mid adolescents with common mental health problems	‘a substantial proportion of mental health problems arise before the age of 14 and changes in adolescent sleep occur around puberty. As such, it is likely that those in early adolescence (i.e., around 11–13) would benefit the most from this intervention as it may prevent both issues from escalating further (e.g., most benefit in terms of early intervention)’ ‘This might be earlier for girls/older for boys, given the gender difference in onset of emergence of difficulty’ ‘Adolescents with anxiety and/or depression, with clinical levels of insomnia’ ‘May work for some… but less well for teens with sleep/wake phase [disorder]’
Content and design of the intervention is appropriate, although may require adaptation	‘Structure and content both appear appropriate for young people with comorbid sleep and mental health problems’ ‘May be worth considering splitting the cognitive and relaxation techniques into two sessions’
Method of delivery	‘to be delivered in whatever way was most helpful for the young person, with facilitation’ ‘ensuring a week between sessions to complete homework activities and reflect on this at the start of the next session’ ‘If online, young people could move through the sessions at their own pace. If in person then the sessions could be delivered weekly and sleep diaries kept between sessions’ ‘Six shorter/bitesize sessions (approx. 30 min)’ ‘Short sessions with very little didactic teaching’
Parent/carer involvement is necessary for implementation	‘Parents are definitely needed to help facilitate the implementation of the techniques’ ‘Evidence suggests parent involvement is crucial but this is tricky to obtain’ ‘Would be helpful for parents to receive resources to support at home implementation and potentially to attend their own session with the facilitator to receive a summary of what the young person has learned’

#### 3.2.1 Sleep Intervention Is Appropriate for Early–Mid Adolescents With Common Mental Health Problems

Four participants commented specifically on the appropriate age of adolescents. Although there was variation in the recommendations, the youngest recommended age was 11 years and the oldest was 15 years. The remaining participants either did not comment or provided a specific age range; instead, they cited ‘adolescents’, ‘young people’ and ‘teens’. Most participants (5/7) indicated that the intervention was suitable for adolescents with common mental health problems (such as anxiety or depression). One participant indicated that the intervention would not be appropriate for adolescents in significant distress who were self‐harming or experiencing suicidal ideation. There were conflicting opinions regarding the appropriateness of the intervention for individuals with delayed sleep/wake phase disorder, with one participant indicating it would be helpful, and another highlighting it would not be helpful for those with ‘greater eveningness’. Therefore, this was included as a follow‐up question in R2.

#### 3.2.2 Content and Design of the Intervention Is Appropriate, Although May Require Adaptation

After reviewing the proposed intervention protocol (see Figure 2), five participants indicated that the content and structure was appropriate as presented. However, three participants provided suggestions for adaptations. Suggestions included splitting the cognitive and relaxation components into two sessions. Notably, two participants enquired about the inclusion of sleep restriction as this was not included in the proposed protocol in R1. Therefore, this was included as a question in R2 to ask the other participants and explore whether they felt it was appropriate to include SRT in the protocol. Engagement and motivation were also highlighted as potential barriers for the delivery of the intervention to adolescents. Consequently, two participants stressed the importance of including active components or activities, expressing that psychoeducation and cognitive techniques may be difficult to deliver to adolescents as ‘this age group likes to “do” things (more so than think about it)’.

#### 3.2.3 Method of Delivery

Participants gave feedback on the order of components as outlined in the proposed protocol. Two participants highlighted that the 1 h, 3‐session format may not be sufficient to cover all content proposed in the protocol. This suggested that an additional session(s) may be useful to ensure all relevant information could be included. However, suggestions for the number of sessions varied; three participants suggested a 6‐session format and other participants suggested 1–3 sessions. In addition, four participants highlighted the benefits of including a booster session to tackle any remaining difficulties. Weekly sessions were favoured by participants, ranging from 30 min to 1.5 h each. Experts provided feedback and suggestions for activities, favouring the active involvement of adolescents compared to didactic teaching. Whilst participants highlighted the benefits and disadvantages of both online and in‐person delivery, they did not communicate a strong preference for either modality.

#### 3.2.4 Parent/Carer Involvement Is Necessary for Implementation

All participants demonstrated that parent/carer involvement was necessary for this population, specifically to support the implementation of techniques at home. However, many highlighted that this would be difficult to implement in practice. Three participants highlighted that it is important to consider the individual relationship between the child and parent, as for some adolescents, parental involvement may ‘exacerbate their mental health issues’. Participants varied in their opinions regarding how parents/carers should be involved, citing both resources or attendance at sessions. Suggestions for content included psychoeducation, a summary of each session and advice on how to support the implementation of techniques at home.

### 3.3 Round 2 (R2) Development of the Questionnaire and Findings

For R2, the questionnaire was developed to clarify issues and follow‐up on key uncertainties that emerged following R1. The questionnaire included mainly closed‐ended questions and some open‐ended questions. Specifically, R2 included follow‐up questions about the inclusion of SRT and SCT. The participants were also asked to review the refined protocol (see Figure 2), to confirm whether a 3‐ or 4‐session format was most appropriate, and if the order of components was suitable for adolescents with comorbid mental health problems. Following feedback from R1, one question specifically explored participants views' on the suitability and appropriateness of the intervention for adolescents with comorbid delayed sleep/wake phase disorder, and to enquire about any potential adaptations required for these individuals. Participants were asked whether it would be appropriate to train other non‐sleep/mental health professionals, such as educational psychologists or teachers, to deliver the intervention. Finally, participants were asked to provide additional feedback about the content to be included in parent/carer resources. For closed‐ended questions, participants were asked to respond using ‘yes’, ‘no’ or ‘I do not have the expertise to respond to this question’ and optional text boxes for additional detail if required. Following completion of R2, the data was downloaded and analysed by the research team using frequency statistics for quantitative data and content analysis for open‐ended questions. The analysis was performed by the same five researchers as in R1. The findings for round 2 are detailed in Table 3.

**TABLE 3:** Round 2 frequency of responses.

	Yes	No	I do not have the expertise to respond
Inclusion of sleep restriction therapy (SRT) and stimulus control (SCT) to the intervention protocol	4 (80%)		1 (20%)
Refined intervention protocol (including SRT & SCT) is appropriate for adolescents with comorbid insomnia and mental health	4 (80%)		1 (20%)
Order of the components is appropriate	4 (80%)		1 (20%)
Four sessions would be more appropriate than three	2 (40%)	2 (40%)	1 (20%)
Appropriate for comorbid delayed sleep/wake phase disorder		1 (20%)	4 (80%)
The intervention would be appropriate for delivery by non‐sleep experts with appropriate training (e.g., teachers, Educational Psychologists)	4 (80%)	1 (20%)	

Due to the lack of response to the question in R2 regarding the appropriateness of the intervention for adolescents with comorbid delayed sleep/wake phase disorder (DSWP), an additional expert with expertise in CBTi was consulted. The expert advised that manualised CBTi may not be appropriate for individuals with DSWP as this would require individual tailoring of key components such as exposure to light. In addition, the expert was consulted about the SRT protocol, specifically about the appropriate minimum time in bed prescription (TIB) for adolescents. They recommended the most appropriate minimum TIB for this population was 6 h because of the greater sleep need in this population. The data from R2 was used to further refine the protocol and intervention materials in preparation for R3.

### 3.4 Intervention Refinement Following Round 2

The intervention protocol and content were finalised based on the findings from R2. Following this, the intervention materials were developed and reviewed by the research team prior to R3. See Table 4 for a summary of the amendments made.

**TABLE 4:** Overview of feedback from expert panel, research team recommendations and final protocol.

	Summary of feedback from expert panel	Research team recommendations following R1 and R2	Final protocol following R2
Target population	Adolescents aged 12–15 years with common mental health problems (e.g., anxiety and depression)	Adolescents should be excluded if they had contraindicators for SRT, and individuals experiencing suicidal ideation	Inclusion: Adolescents 12–15 years with comorbid mental health problems who have had no previous experience of CBTI Exclusion criteria: Adolescents with contraindicators for SRT (seizure conditions or any condition that is exacerbated by sleep deprivation), experiencing suicidal ideation
Content and components	Inclusion of SRT and SCT	Inclusion of SRT and SCT	All components of CBTi included in intervention protocol and materials. For SRT, the minimum TIB prescription was 6 h to account for additional sleep need in adolescents
Mode of delivery	3–6 sessions, delivered weekly by a trained facilitator	4 sessions are feasible, delivered weekly, by trained practitioners within a mental health team	4 weekly sessions, delivered in groups or individually, by trained practitioners within a mental health team
	Parent/carer involvement is necessary—either attendance at sessions or resources	Parent/carer involvement is necessary—preference for online resources to minimise burden on practitioners, time and resources. To include session summary and advice for implementation	Parent/carer videos to support at‐home implementation of each session

Abbreviations: SCT, stimulus control therapy; SRT, sleep restriction therapy.

### 3.5 Round 3 (R3) Questionnaire and Findings

In R3, each of the session materials were presented and participants were asked to respond to ‘Is the content and presentation of session X appropriate for delivery to adolescents attending Mental Health Services?’ Participants were asked to respond using ‘Yes, it is appropriate’, ‘It is appropriate but requires additional adaptations (please use the space below to explain your answer)’, or ‘No, it is not appropriate (please use the space below to explain your answer)’.

Despite several attempts to contact the previous participants, only one participant responded in R3. This individual reviewed the final intervention materials. Following their review, there were no new recommendations or changes made to the protocol or materials, except for the correction of a spelling error. The final intervention package includes the following materials: 4 PowerPoint presentations and accompanying parent/carer videos, a participant workbook, digital sleep diary and delivery manuals.

## 4 Discussion

The aim of this study was to use an expert consultation process to develop a CBTi intervention protocol and materials for adolescents with insomnia and comorbid mental health problems. The objectives of this study were to address key gaps in the literature (Meltzer et al. 2021; Reynolds et al. 2023) by defining the appropriate target population, CBTi treatment components, mode of delivery and family involvement. We also worked closely with a mental health service (through members of the research team) to design the protocol to ensure it was feasible for delivery in practice.

The first objective of the study was to identify the appropriate target population by exploring experts views on the appropriateness of the CBTi intervention for adolescents with insomnia and mental health problems. The experts indicated that the intervention would be particularly beneficial for early–mid adolescents. This is likely because this age group is affected by changes to the sleep bioregulatory systems, which can predispose them to insomnia (Carskadon 2011). In addition, the findings suggest the intervention would be appropriate for adolescents with mental health problems, such as anxiety and depression. This is in line with previous studies that show CBTi is effective for improving symptoms of depression and anxiety in adolescents (Åslund et al. 2020; Blake et al. 2017). In R1, one participant indicated that it may not be appropriate for individuals with delayed‐sleep wake phase syndrome. Whilst this was included as a follow‐up question in R2, most participants (4/5) indicated that they did not have the expertise to comment. Therefore, an additional expert was consulted who advised it may not be suitable for delivery by non‐sleep experts as this would require additional, individual assessment and specific tailoring of components, which is consistent with other reports in the literature (Crowther et al. 2023; Evans and Hasler 2022). The intervention was designed for delivery by trained mental health practitioners who do not have the sleep expertise that would be required to tailor components. Training non‐sleep specialists to deliver the intervention is central to improving access to effective treatment for insomnia within adolescent mental health services. Future research could explore this more fully and include participants with specialised expertise in managing circadian disruption to appropriately advise which techniques should be integrated into the protocol.

The second objective was to examine the appropriateness of the intervention content. The majority of participants in R1 indicated that the proposed intervention protocol was acceptable and appropriate, although some adaptations and additional components were required. All participants (with appropriate expertise) indicated that SRT should be added to the protocol. This is further supported by a number of previous trials that have utilised SRT with adolescents and reported improvements in insomnia severity, sleep duration and quality (Åslund et al. 2020; Cliffe et al. 2020; Mathews et al. 2023; Orchard et al. 2020; Zetterqvist et al. 2021). Although some other studies have included a milder form of SRT for adolescents with comorbidities, with a focus on sleep scheduling or amending wake‐up times (Bradley et al. 2018; Rollinson et al. 2021, 2024). There are currently no standard guidelines for the delivery of CBTi for adolescents. This is problematic, particularly for the delivery of SRT and the minimum TIB prescription, because adolescents have a greater sleep need compared to adults (Paruthi et al. 2016). Therefore, following a literature review of the previous intervention studies and consultation with an SRT expert, it was decided by the research team that a minimum TIB prescription of 6 h would be appropriate to account for the sleep need in this population (de Bruin et al. 2018).

Some highlighted that motivation and engagement are typical barriers to behavioural change interventions in this population. Therefore, each session was designed to comprise limited didactic teaching, with an emphasis on behavioural change activities (such as goal setting, planning and behavioural experiments), accompanied by at‐home implementation of techniques. All participants highlighted that parent/carer involvement was necessary to support motivation and engagement. Additionally, it has been reported that family support is a good protective factor for good sleep health in adolescents (Maratia et al. 2023). Therefore, parent/carer videos were created to provide a summary of each session, information about the weekly tasks as well as advice on how to support at‐home implementation.

The final objective was to consult with the participants to explore their views on the most appropriate method of delivery (i.e., mode, number and order of sessions, facilitator). Within the panel, there was no strong preference for in‐person or online delivery; however, as a research team, it was decided that in‐person delivery to groups or individuals would be most appropriate for delivery in a mental health service. Previous research has demonstrated comparative effects of CBTi regardless of whether it is delivered individually, remotely, or in groups (Simon et al. 2023). Some participants in R1 highlighted that the 3‐session format may not be sufficient to cover all of the content and suggestions ranged from 3 to 6 sessions. Whilst the decision to include four sessions was informed by the expert participants insights, it was a pragmatic decision made by the research team as it was the most feasible option for delivery in a mental health setting. A study by Edinger et al. (2007) reported that a 4‐session CBTi format was optimal compared to 6–8 session protocols. Furthermore, in R2, all participants with relevant expertise indicated that the proposed order of sessions was appropriate. To our knowledge, there is no evidence to suggest that an order‐effect exists for the delivery of CBTi. Lastly, the participants highlighted that the intervention should be delivered by a trained facilitator, with most participants in R2 (4/5) indicating that it would be appropriate to train non‐specialist practitioners to deliver the intervention. Recent research has demonstrated the effectiveness and feasibility of this approach (Kyle et al. 2023; Rollinson et al. 2021).

To our knowledge, this is the first study to utilise an iterative expert‐consultation methodology to develop a manualised CBTi intervention for adolescents with insomnia and comorbid mental health problems. This is important, as stakeholder involvement is crucial to enhance the feasibility of interventions and increase the chances of integration and delivery beyond the initial research protocol. Increasing access to sleep training and interventions is beneficial to increase awareness and knowledge of sleep problems amongst practitioners and improve access to evidence‐based sleep interventions (Baglioni et al. 2023; Jernelöv and Blom 2023).

However, there are some key limitations to consider. While we initially intended to conduct a formal Delphi study (King et al. 2021), the limited sample size meant that we were unable to consider this study a true Delphi and instead refer to it as an expert‐consultation approach throughout. There was an attempt to recruit a larger number of participants, but the response (39% in R1) and retention rates (28% in round 2, 6% in round 3) were particularly low. This may be attributed to the long duration between and during the rounds that can lead to diminished interest or engagement from experts. There is a lack of consensus on the ideal sample size for Delphi research, but panels ranging from 8 to 23 experts are typically considered optimal (Shang 2023). Despite this, several key elements of Delphi were upheld—including the iterative nature, anonymity of responses and consensus; thus, the findings provide valuable insights. The intervention protocol and materials were designed and reviewed by the research team that comprised behavioural sleep medicine experts and a CAMHS Head of Service with extensive experience and expertise in CBTi. Therefore, although the panel consisted of only seven experts (and 1 in R3), there were insights from an additional three experts via the research team (L.F., M.C. and K.M.). Whilst expert consultation is beneficial, it does offer a lower level of evidence as it relies on expert opinion, and there are no standardised universal guidelines for conducting this type of research. However, this method can be particularly useful to improve and inform decision‐making in both clinical and research contexts, particularly when there is a lack of clear guidance, as is the case with CBTi for adolescents (King et al. 2021). A final limitation is that the study did not involve any adolescents with lived experience of comorbid insomnia and mental health problems. The aim of this study was to develop an evidence‐based intervention, informed by expert views and recommendations for best practice. Therefore, the next stage of this project will be to pilot the intervention and examine the acceptability to adolescents, parents/carers and practitioners. This will inform any additional refinements before a full evaluation trial is conducted.

## Author Contributions

**Stephanie McCrory:** conceptualization, formal analysis, investigation, methodology, project administration, resources, visualization, writing – original draft, writing – review and editing. **Megan Crawford:** conceptualization, methodology, supervision, writing – review and editing. **Kenneth MacMahon:** conceptualization, methodology, supervision. **Carey Ross:** formal analysis, resources, methodology. **Dipalika Mohanty:** formal analysis, methodology, resources. **Dinaish Mistry:** formal analysis, methodology, resources. **Anastasia Thalia Fulton Chadwick:** methodology, formal analysis, resources. **Leanne Fleming:** conceptualization, methodology, supervision, writing – review and editing.

## Conflicts of Interest

The authors declare no conflicts of interest.

## Supporting information


**Data S1:** Supporting Information.

## Data Availability

The data that support the findings of this study are available from the corresponding author upon reasonable request.
