# Supplementary material for: Developing a Cognitive Behavioural Therapy for Insomnia Intervention for Adolescents With Co‐Morbid Mental Health Using an Iterative Expert Consultation Process
Source: J Sleep Res. 2025 Aug 20;35(2):e70174. doi: 10.1111/jsr.70174 (PMC13003277; doi:10.1111/jsr.70174)
Supplement: Supplementary file 1 — Data S1: Supporting Information. [file JSR-35-e70174-s001.docx]

# **Supplementary file:**

**Demographic questions**

Please select your age from the categories below:

- 18-24 years old
- 24-34 years old
- 35-44 years old
- 45-54 years old
- 55-64 years old
- Above 65 years old

How would you describe yourself?

- Female
- Male
- Non-binary / third gender
- Prefer to self-describe ____________
- Prefer not to say

Where are you located?

- Europe
- North/Central America
- South America
- Africa
- Asia
- Other, please specify ____________
- Prefer not to say

Please select the highest level of education you have attained.

- Doctorate degree
- Masters degree
- Bachelor’s degree
- Associate degree
- Other, please specify ____________
- Prefer not to say

Please select your current employment status (Please select all that apply)

- Employed full-time (please insert your job title)
- Employed part-time (please insert your job title) ________
- Self-employed / freelance (please insert your job title) ___________
- Full-time student
- Part-time student
- Retired (please insert your previous job title) ___________
- Unemployed (please insert your previous job title) ___________
- Other, please specify
- Prefer not to say

Please select the role that best describes you (Please select all that apply)

- Academic
- Practitioner
- Both
- Other, please specify ____________

Please select the specialism that best describes you from the list below. (please select all that apply)

- Adolescent behavioural sleep medicine
- Adolescent mental health
- Other, please specify ____________

**Round 1 questionnaire:**

1. **In your opinion, who would benefit most from a targeted intervention to improve adolescent sleep and co-morbid mental health?**

The intervention protocol currently includes the following components and content:

| **Session** | **Component** | **Content** |
| --- | --- | --- |
| **1** | **Psychoeducation** | Why we sleep |
|  |  | How we sleep |
|  |  | Factors that affect sleep |
| **2** | **Sleep Hygiene and stimulus control** | Diet |
|  |  | Caffeine |
|  |  | Substance Use |
|  |  | Exercise |
|  |  | Bedroom/sleep environment |
|  |  | Sleep scheduling |
| **3** | **Cognitive & Relaxation techniques** | Understanding thoughts and emotions |
|  |  | Types of thoughts |
|  |  | Thought blocking |
|  |  | Racing mind |
|  |  | Thought restructuring |
|  |  | Putting the day to rest Guided imagery Breathing exercises Progressive Muscle Relaxation |

1. **Please comment on the structure and content of the sleep intervention (outlined above) and whether you feel it is appropriate for adolescents with co-morbid sleep and mental health difficulties?** If you feel that you do not have the expertise to provide feedback in this section, please leave this question blank.
2. **What (if anything) is missing from this intervention?**
3. **What is the most appropriate delivery of a sleep intervention to adolescents with co-morbid sleep and mental health difficulties?**
4. **Do you feel parental/carer input is appropriate for an adolescent sleep intervention?**

- **Yes**
- **No**

1. **If yes, please outline the type of parental/carer involvement that you think would be helpful**
2. **If no, please explain why.**

**Round 2 questionnaire**

1. **Based on the feedback we received in round one, and the evidence base, we have now added Sleep Restriction Therapy to our protocol, do you think this is appropriate for adolescents?**

- Yes (if selected Protocol 1 was displayed)
- No (if selected Protocol 2 was displayed)
- I do not have the expertise to respond to this question (if selected question 5 was displayed)

**Protocol 1:**

We have added sleep restriction therapy to the protocol below. 
Each session will be delivered for approximately 1 hour each week.

| **Session** | **Component** | **Content** |
| --- | --- | --- |
| 1 | **Psychoeducation,**  **Sleep Hygiene &**  **Sleep Restriction Therapy** | Why we sleep  How we sleep  Factors that affect sleep  Good sleep hygiene practices  Sleep restriction therapy |
| 2 | **Relaxation &**  **Stimulus control** | Guided imagery  Breathing exercises  Progressive Muscle Relaxation  Stimulus control  Sleep restriction titration |
| 3 | **Cognitive** | Understanding thoughts and emotions  Types of thoughts  Thought blocking  Racing mind  Thought restructuring  Sleep restriction titration |

**Protocol 2:**

Based on the feedback from round 1, we have now amended the protocol below. 
Each session will be delivered for approximately 1 hour each week.

| **Session** | **Component** | **Content** |
| --- | --- | --- |
| **1** | **Psychoeducation &**  **Stimulus control** | Why we sleep  How we sleep  Factors that affect sleep  Good sleep hygiene practices  Stimulus control |
| **2** | **Sleep hygiene &**  **Relaxation** | Good sleep hygiene practices  Guided imagery  Breathing exercises  Progressive Muscle Relaxation |
| **3** | **Cognitive** | Understanding thoughts and emotions  Types of thoughts  Thought blocking  Racing mind  Thought restructuring |

1. **Do you think this protocol is appropriate for delivery to adolescents attending mental health services with co-morbid insomnia and mental health?**
2. **Do you think the 3 session format (outlined above) is appropriate, or should we add an additional session?**

- 3 session format is appropriate
- 4 session format would be more appropriate

1. **Do you think the order of components and content (outlined above) is appropriate?**

- Yes
- No (if selected question 4.1 displayed)
  1. **What amendments should be made to the order of components and content (outlined above) to ensure that it is appropriate?**

     Please drag and drop the components in to each section to re-order the components.


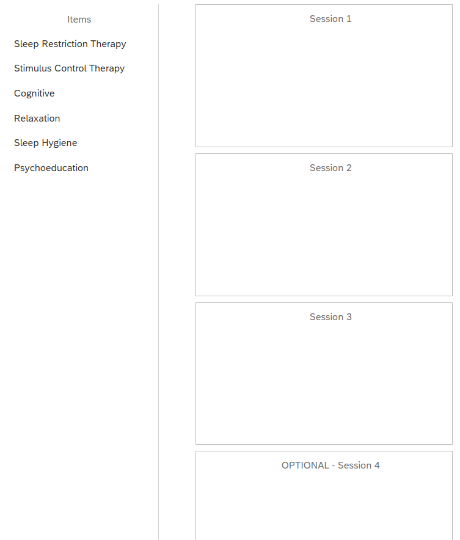


1. In round 1, we received some feedback that the intervention would not be appropriate for adolescents with co-morbid Delayed Sleep/Wake Phase Disorder. Based on this feedback, we are interested to consider whether adolescents with Delayed Sleep/Wake Phase Disorder should be excluded from participating in the intervention. 
   **In your opinion, is it appropriate to deliver the intervention to adolescents with co-morbid insomnia and Delayed Sleep/Wake Phase Disorder?**

- Yes, I think it is appropriate to include these individuals (if selected, question 5.1 was displayed)
- No, I do not think it is appropriate to include individuals
- I do not have the expertise to respond to this question (if selected question 6 was displayed)
  1. **Can the intervention be delivered in the same way to individuals with co-morbid insomnia and Delayed Sleep/Wake Phase Disorder?**
- Yes _____________________
- No _____________________

1. **Do you think that this intervention could be delivered, with appropriate training, by Educational Psychologists or school teachers? Please use the space below to explain your answer.**
2. **What information would be most useful to include in a video resource for parents/carers in order to maximise the likelihood that the young person will engage with the intervention and at-home tasks?**

**Round 3 questionnaire**

**Session materials presented individually.**

1. **Is the content and presentation of session 1 appropriate for delivery to adolescents attending Mental Health Services?**

- Yes, it is appropriate
- It is appropriate but requires additional adaptations (please use the space below to explain your answer)
- No, it is not appropriate (please use the space below to explain your answer)

1. Please use this space to insert any additional comments/feedback.
